# Medical and Interventional Therapy for Spontaneous Vertebral Artery Dissection in the Craniocervical Segment

**DOI:** 10.1155/2017/7859719

**Published:** 2017-02-22

**Authors:** Guiyun Zhang, Zuoquan Chen

**Affiliations:** ^1^Department of Neurosurgery, Shanghai Jiaotong University First People's Hospital, Shanghai 200080, China; ^2^Department of Neurosurgery, Tenth People's Hospital of Tongji University, Shanghai 200072, China

## Abstract

*Background and Purpose.* Spontaneous vertebral artery dissection (SVAD) is an important reason for posterior-circulation-ischemic stroke in the young and middle-aged population. Although some previous reports reveal a favorable outcome with conservative therapy, it is still controversial in the treatment of SVAD in some specific patients. Herein, we present our 10 years of clinical experience for SVAD at this location.* Material and Methods.* 20 patients with 20 SVADs in V2 and V3 segments were retrospectively studied. Clinical manifestations and imageology materials were collected and analyzed. All the patients underwent anticoagulation except for one patient because of contraindication. 14 patients underwent Wingspan stents implantation with general anesthesia.* Results.* In our sample, ischemia (infarction or transient ischemic attack, TIA) was found in all the patients. Angiographic stenosis and dissection aneurysm were the most common findings in the segments mentioned above. 19 of the patients (95%) got the excellent imageological and clinical outcomes.* Conclusions.* According to our experience in this group, although anticoagulation is effective in vertebral artery dissection, interventional therapy for SVADs in V2 and/or V3 segments is preferred in some specific patients. Stent with higher radial supporting and flexibility, such as Wingspan stent, is suggested.

## 1. Introduction

Spontaneous vertebral artery dissection (SVAD) is a rare condition that occurs in the young and middle-aged population with an estimated annual incidence between 1 and 1.5 per 100,000 [1, 2]. The typical symptoms are ischemia and/or subarachnoid hemorrhage (SAH), which were usually seen in the extracranial and intracranial segment, respectively [3, 4]. SVAD is most commonly observed in the V2 and V3 segments of the vertebral artery (VA) [5, 6]. Although connective tissue abnormalities, hyperhomocysteinemia, fibromuscular dysplasia, and hypertension are factors associated with SVAD, the tortuous and a relative large range of motion of VA at craniocervical level are perhaps the major predisposing factors. As the greatest risk of stroke in craniocervical dissections appears to occur in the first few weeks [7], it is very important to treat these patients with effective methods in time. Although medical therapy has shown a favorable outcome, there are still more than 10% of the patients with serious disability and even death [8]. Until now, few studies involve in clinical relapse and imageological characteristics. Despite the lack of standard guidelines for the treatment of SVADs in V2 and V3 segments, it is the common practice to treat aggressively patients who present with repeated relapse of ischemia during conservative treatment with maximum of anticoagulation. The purpose of this study was to describe the characteristics of clinic and imageology in these specific patients and identify the safety and efficacy of Wingspan stent in the treatment of SVAD in V2 and V3 segments as an alternative method.

## 2. Materials and Methods

20 patients with SVADs in the V2 and V3 segments confirmed by digital subtract angiography (DSA) were retrospectively analyzed between May 2004 and April 2014 at our center. There was only one patient diagnosed as fibromuscular dysplasia presenting with the stenosis of left carotid artery and dissection of left VA, who was treated conservatively, because the medical therapy was effective. And the six patients with hypertension belonged to the two groups (conservative and interventional groups) equally. There were no histories of other risk factors, such as connective tissue abnormalities and hyperhomocysteinemia. Pre- and postoperative neuroimaging were studied (including magnetic resonance angiography, MRA, DSA, and/or magnetic resonance imaging, MRI). Patients with SVADs in the segment of V1 and V4 (intracranial segment) were excluded. The diagnosed standards of SVADs on DSA included (1) irregular stenosis of VA; (2) double lumens; (3) intimal flap; (4) string sign; (5) string beads sign; (6) aneurysmal dilation; (7) all these patients who did not suffer from trauma or iatrogenic injury. All the 20 patients were followed up with DSA ranging from 6 to 60 months (mean term 28.6 months). The descriptive statistic methods (mean value, percentage, and standard deviation) were used in this study.

### 2.1. Patients' Characteristics and Imageology Findings

Computed tomography (CT) and MRI were performed in all the 20 patients (6 females, 14 males; mean age: 37.05 years, range: 23–46 years) presenting with acute ischemia in the posterior circulation (pons, cerebellum, and/or medulla), and DSA was used for following up. The associated signs and symptoms included cerebellar ataxia (*n* = 12), nystagmus (*n* = 15), dysphagia and dysarthria (*n* = 1), tinnitus (*n* = 10), vomiting (*n* = 8), neck pain (*n* = 6), and headache in ipsilateral occiput (*n* = 8). Clinical grading and outcome of following up (6 months after the initial treatment) were evaluated by National Institute of Health stroke scale (NIHSS) and modified Rankin score (mRS), respectively. The patients' characteristics, dissection location, vertebral artery dominance, and imageology findings were listed on Tables 1 and 2.

### 2.2. Medical and Interventional Therapy

Six of the patients underwent a usual treatment with intravenous heparin followed by oral warfarin with a target international normalized ratio (INR) of 2.5 (range, 2.0–3.0) for 3 months. Although under the maximal dosage of anticoagulation with INR of 3.0 for 14 days, 13 of the patients underwent stents implantation with the preparation of double-antiplatelets (300 mg aspirin and 75 mg clopidogrel oral daily) as an alternative because of the relapse of stroke and the persistent positive signs and symptoms (the specific evaluation criteria and definition of “the relapse of stroke and the persistent positive signs and symptoms” in this study referred to symptomatic patients who suffered from SVADs with the treatment of medicine for at least two weeks, and the positive signs (cerebellar ataxia, nystagmus, dysphagia, and dysarthria,) and symptoms (tinnitus, neck pain, and headache) were persistently existing or even worse, or other neural dysfunction appeared). One of the patients did not receive anticoagulation for the multiple and extensive infarcts in the cerebellum and occipital lobe followed by a secondary SAH and ventricle hemorrhage. External ventricular drainage was performed in this patient for obstructive hydrocephalus and then followed by stenting 4 weeks later (the eclectic dosages of aspirin (100 mg) and clopidogrel (25 mg) were adopted 3 days before procedure and maintained for 3 months, and then the clopidogrel was stopped, and the aspirin was still going on. During the first week after stenting and the 3 days before procedure, we monitored the coagulating and platelet functions consecutively). For the patients with stenting, double-antiplatelets were needed at least 3 days before and 3 months after the procedures, then with a 100 mg oral aspirin per day. On the day of procedure, a bolus of 2500 U of heparin was administered. During the duration of procedure, drip infusion through the 6 F-guiding catheter of 1000 U of heparin was administered continuously. Activated clotting time was controlled between 250 and 300 seconds. Stent implantations, under general anesthesia, were performed on a biplane angiographic unit (LCN+, GE company, US). SL-10 microcatheter (Stryker, US) was used to facilitate the guider wire to go across the lesion of the involved VA. The gateway balloon and Wingspan self-expandable stent (Stryker, US) were chosen according the artery diameter and length of the involved artery. For the treatment in subacute phase, predilatation with gateway balloon was preferred in order to diminish the residual stenosis and get an anatomical healing.

## 3. Results

In our sample, men were more frequently affected than women (14 : 6), and the mean age was 37.05 years. Ischemia (infarction or transient ischemic attack, TIA) was found in all the patients (infarction, 17; TIA, 3). Subarachnoid and ventricle hemorrhage secondary to the infarction were seen in one. Eleven dissections were found in the V2 segments and 9 in V3. Angiographic stenosis (12/20) and dissection aneurysm (6/20) were the most common findings in the segments of V2 and V3, and the last was intimal flap (2/20). Unilateral VA dominance was found in 15 patients and VA equability in the others. Twelve lesions were on the dominant VA and three in the nondominant. Reangiography showed that two patients had extension of the dissections from V2 to V3 segment on the day of procedures. The average term of following up by angiography was 28.6 months (ranging from 6 to 60 months) in all of the patients. Nineteen of the patients (95%) got the excellent imageology and clinical outcomes (mRS = 0). The patient with subarachnoid and ventricle hemorrhage also had a good recovery (mRS = 1).

### 3.1. Technical and Clinical Results

Fourteen patients (six dissections in the V2 segment and eight in V3 initially), twelve lesions on the dominant and two on the nondominant VA, underwent stent implantation successfully. Among the six dissections in the V2 segments, two were found to extend from V2 to V3 segment before stenting. There was no procedure related complication. Eighteen dissections were predilated through gateway balloon. Remnant stenosis (<10%, the initial stenosis rate was 80–95%) presented in one (7%). Thirteen of the fourteen patients got a satisfactory outcome without any residual stenosis and none of them presented with relapse of ischemia or positive symptoms and signs. The one with subarachnoid and ventricle hemorrhage also had a relative good recovery since the stent implantation. No case of hemorrhage was seen. Six to sixty months of following up showed that there was no in-stent stenosis, and the residual stenosis after the first stenting disappeared. None of them presented fresh infarct on MRI.

## 4. Discussion

This is the first study of SVAD in the craniocervical segment treated with medicine combined with self-expandable stent. Previous study has showed that the conservative therapy including anticoagulation and antiplatelet is effective for SVADs, especially for those in the extracranial segment [4, 5]. But according to the ten years of our experience about this kind of disease, it is not always the case.

**Figure 1:**
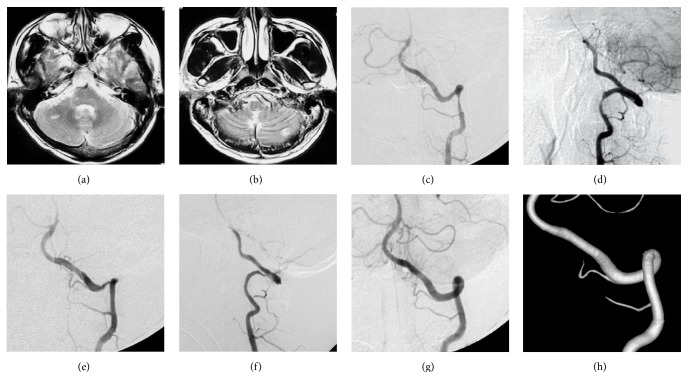
MRI ((a) and (b)) showed multiple infarcts in the bilateral cerebellum especially in the right cerebellar hemisphere. Frontal and lateral DSA ((c) and (d)) of left VA angiogram showed the dissection in the segment of V3. Stenosis was the major appearance. Although the left VA was the nondominant one, the embolus is easy to escape and enter into both the right and left AICA (more into the right one) due to neck motion and the characteristics of anatomy of right AICA (the VA distal to the dissection, inferior segment of BA, and the initial segment of right AICA are almost in a line). Frontal and lateral DSA showed the stenosis disappeared after stenting ((e) and (f)). 2-dimensional and 3-dimensional DSA ((g) and (h)) showed the anatomical healing of the involved VA when following up. MRI: magnetic resonance image; DSA: digital subtraction angiography; VA: vertebral artery; AICA: anterior inferior cerebellar artery; BA: basilar artery.

**Figure 2:**
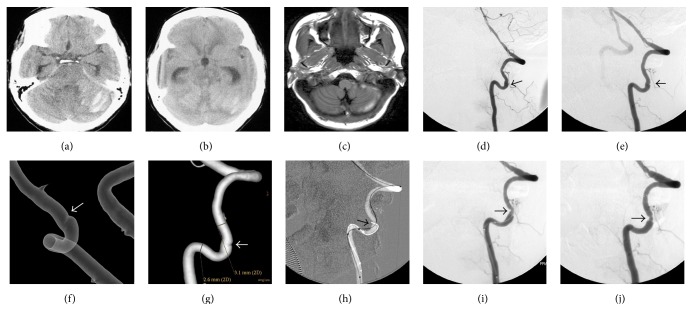
CT (a) showed lamellar infarct in the pons and worm of cerebellum and SAH. CT (b) showed obstructive hydrocephalus. MRI (c) showed SAH gradually decreased after ventriculoperitoneal shunt. Lateral and oblique DSA ((d) and (e)) of left VA showed the dissection in the segment of V2. Transparency and volume rendering ((f) and (g)) showed the intimal flap. It is the major appearance. During the procedure, the micro-guide wire was difficult to pass through the true lumen until the microcatheter was used (h and i). Lateral DSA showed slight remnant stenosis after stenting (j). CT: computed tomography; MRI: magnetic resonance image; DSA: digital subtraction angiography; VA: vertebral artery.

In the samples of ours, fourteen patients (14/20) underwent stent implantations finally, because the anticoagulation or antiplatelet did not bring any efficacy. Although with a normative medical therapy, it was difficult to stop the relapse of stroke and eliminate the symptoms. So we had to use stents for these patients in order to recover the continuity of VA and diminish defluxion of thrombus. In the fourteen patients, there were twelve lesions on the dominant VA, and eight patients of them had no development of contralateral VA distal to the orifice of posterior inferior cerebellar artery (PICA). Stenosis caused by the dissection brought a catastrophic ischemia in the poster circulation without sufficient collateral compensation. Angiography of the left two patients with lesions on the nondominant VA showed the dissection-pattern and anatomy characteristics, which was associated with cerebellar infarction (Figures 1 and 2).

SVADs in the craniocervical segment are much more than those in the other locations. The certain reasons are not well known, but the relative extensive range of motion and tortuosity of VA has been thought as the usual cause. Hemodynamic and mechanical motion might be the reasons for the difficulty of dissection healing and even cause the dissection to extend forward into the distal part. In our samples, two patients with dissections in the segment of V2 were found to deteriorate on the angiography. The extensive dissection further aggravated the hemodynamic ischemia. This phenomenon, in some degree, might present an explanation for inefficacy of conservative treatment.

Previous study indicated that embolism was usually considered as the major reason (90%) for ischemia (stroke, TIA) [9–11], and the anticoagulation or antiplatelet therapy was effective for the majority of the patients. But in our group, only six patients (6/20) were treated conservatively and got the comparative good results. The left fourteen patients had to receive stent implantations because of continuous aggravation of the symptoms, even under a maximum of anticoagulation. The different outcome between our study and the previous was associated with asymmetry of VA and insufficient collateral compensation. For the fourteen patients with stenting, hemodynamic ischemia was the main reason in eight whose lesions were in the dominant VA with the contralateral nondevelopment of VA distal to the orifice of PICA. Embolus escaping from the dissection was the other probable reason in the left six patients (two lesions in the nondominant VA and four in dominant VA).

The fact that hemodynamic ischemia and embolisms may occur in the same patients has to be kept in mind in clinical practice. When anticoagulation is not effective, stenting should be considered as an alternative method. In De Bray et al.'s prospective study of 22 consecutive vertebral artery dissections [12], about 50% of involved vertebral arteries were not found recanalized. For the young patients, to save the involved vessel is very important. When making the treatment strategy of SVAD, the dominance of VA, relationship between dissection and dominant VA, collaterals (patient's posterior circulation is supported by only a dissected vertebral artery without posterior communicating artery, and the contralateral vertebral artery development is poor), anatomy characteristics, and pattern of dissection should be analyzed adequately. Stenting as an alternative method should be suggested aggressively when the conservative therapy could not bring any further improvement. Fourteen days later, the endovascular therapy should be carried out if the conservative treatment does not work. Those who suffered from connective tissue abnormalities in progress were not suitable for stenting. When stenting is needed the Wingspan self-expandable stent is preferred as its characteristics of higher radial supporting and flexibility. Because of the rare incidence of this kind of lesion, single-center-study really has the shortcoming in sample size. We look forward to a further multi-center-study in the future.

## Figures and Tables

**Table 1:** Summary of characteristics of the 14 patients with SVAD treated by stenting.

Patient	Sex	Age	HT	FHS	DM	Signs/symptoms	SVAD location	VA dominance	Devel of con-VA	NIHSS	mRS	Follow-up (months)
1	m	39	−	+	−	A/N/T/V/H	V3(L)/DoVA(−)	+	+	2	0	6
2	m	38	+	−	−	A/N/V/P	V3(R)/DoVA(+)	+	−	1	0	12
3	m	41	−	−	−	N/T/V/P	V2(L)/DoVA(+)	+	+	1	0	9
4	m	29	−	−	+	A/N/V	V2(R)/DoVA(+)	+	−	1	0	36
5	m	46	−	−	−	A/N/T/V	V3(R)/DoVA(+)	+	−	1	0	24
6	m	38	−	−	−	A/N/V/P	V3(L)/DoVA(+)	+	+	1	0	36
7	m	45	+	−	−	A/N/T/H	V2(L)/DoVA(+)	+	−	1	0	60
8	f	33	−	−	−	D&D	V3(L)/DoVA(+)	+	−	3	1	48
9	m	37	−	−	−	A/N/T/V	V3(R)/DoVA(+)	+	+	1	0	48
10	m	40	−	−	−	A/N/P	V2(L)/DocVA(−)	+	+	1	0	30
11	f	46	+	−	−	A/N/T/H	V3(R)/DoVA(+)	+	−	1	0	24
12	m	28	−	−	−	A/T/H	V2(L)/DoVA(+)	+	−	1	0	30
13	m	35	−	−	+	A/N/T/H	V3(L)/DoVA(+)	+	+	1	0	36
14	m	36	−	−	−	A/V/H	V2(L)/DoVA(+)	+	−	1	0	48

A: ataxia; N: nystagmus; T: tinnitus; V: vomiting; H: headache; P: neck pain; D&D: dysphagia and dysarthria; m: male; f: female; SVAD: spontaneous vertebral artery dissection; VA: vertebral artery; Devel of con-VA: development of contralateral vertebral artery distal to the orifice of PICA (posterior inferior cerebellar artery); NIHSS: National Institute of Health stroke scale; mRS: modified Rankin score; V3: the third segment of vertebral artery; V2: the second segment of vertebral artery; L: left; R: right; DoVA(+): dissection on the dominant vertebral artery; DoVA(−): dissection on the nondominant vertebral artery; HT: hypertension; FHS: family history of stroke; DM: diabetes mellitus.

**Table 2:** Summary of characteristics of the 6 patients with SVAD treated conservatively.

Patient	Sex	Age	HT	FHS	DM	Signs/symptoms	SVAD location	VA dominance	Devel of con-VA	NIHSS	mRS	Follow-up (months)
1	f	37	−	−	−	N/H	V3(L)/DoVA(−)	+	+	0	0	30
2	m	39	+	−	−	N	V2(R)	−	+	0	0	24
3	f	46	+	−	−	N/T	V2(L)	−	+	0	0	36
4	f	27	−	−	−	P/H	V2(R)	−	+	0	0	12
5	f	23	−	−	−	N/T	V2(R)	−	+	0	0	11
6	m	38	+	+	−	P	V2(L)	−	+	0	0	12

N: nystagmus; T: tinnitus; H: headache; P: neck pain; m: male; f: female; SVAD: spontaneous vertebral artery dissection; VA: vertebral artery; Devel of con-VA: development of contralateral vertebral artery distal to the orifice of PICA (posterior inferior cerebellar artery); NIHSS: National Institute of Health stroke scale; mRS: modified Rankin score; V3: the third segment of vertebral artery; V2: the second segment of vertebral artery; L: left; R: right; DoVA(−): dissection on the nondominant vertebral artery; HT: hypertension; FHS: family history of stroke; DM: diabetes mellitus.

## References

[1] Kristensen B., Malm J., Carlberg B. (1997). Epidemiology and etiology of ischemic stroke in young adults aged 18 to 44 years in Northern Sweden. *Stroke*.

[2] Schievink W. I. (2001). Spontaneous dissection of the carotid and vertebral arteries. *New England Journal of Medicine*.

[3] Saeed A. B., Shuaib A., Al-Sulaiti G., Emery D. (2000). Vertebral artery dissection: warning symptoms, clinical features and prognosis in 26 patients. *Canadian Journal of Neurological Sciences*.

[4] Gottesman R. F., Sharma P., Robinson K. A. (2012). Clinical characteristics of symptomatic vertebral artery dissection: a systematic review. *Neurologist*.

[5] Arnold M., Bousser M. G., Fahrni G. (2006). Vertebral artery dissection: presenting findings and predictors of outcome. *Stroke*.

[6] Mokri B., Houser O. W., Sandok B. A., Piepgras D. G. (1988). Spontaneous dissections of the vertebral arteries. *Neurology*.

[7] Biousse V., D'Anglejan-Chatillon J., Touboul P.-J., Amarenco P., Bousser M.-G. (1995). Time course of symptoms in extracranial carotid artery dissections: a series of 80 patients. *Stroke*.

[8] Beletsky V., Nadareishvili Z., Lynch J., Shuaib A., Woolfenden A., Norris J. W. (2003). Cervical arterial dissection: time for a therapeutic trial?. *Stroke*.

[9] Droste D. W., Junker K., Stögbauer F. (2001). Clinically silent circulating microemboli in 20 patients with carotid or vertebral artery dissection. *Cerebrovascular Diseases*.

[10] Srinivasan J., Newell D. W., Sturzenegger M., Mayberg M. R., Winn H. R. (1996). Transcranial Doppler in the evaluation of internal carotid artery dissection. *Stroke*.

[11] Koennecke H.-C., Trocio S. H., Mast H., Mohr J. P. (1997). Microemboli on transcranial Doppler in patients with spontaneous carotid artery dissection. *Journal of Neuroimaging*.

[12] De Bray J. M., Penisson-Besnier I., Dubas F., Emile J. (1997). Extracranial and intracranial vertebrobasilar dissections: diagnosis and prognosis. *Journal of Neurology Neurosurgery and Psychiatry*.

